# Metagenomic analysis of the faecal microbiota and AMR in roe deer in Western Pomerania

**DOI:** 10.1038/s41598-025-93602-4

**Published:** 2025-03-18

**Authors:** Nele Lechleiter, Judith Wedemeyer, Anne Schütz, Julia Sehl-Ewert, Katharina Schaufler, Timo Homeier-Bachmann

**Affiliations:** 1 Institute of Epidemiology, Friedrich-Loeffler-Institut, Südufer 10, 17493 Greifswald, Island of Riems Germany; 2 Department of Experimental Animal Facilities and Biorisk Management, Friedrich-Loeffler-Institut, Südufer 10, 17493 Greifswald, Island of Riems Germany; 3 Epidemiology and Ecology of Antimicrobial Resistance, Helmholtz Institute for One Health, Fleischmannstraße 42, 17489 Greifswald, Germany; 4 University Medicine Greifswald, Fleischmannstr. 8, 17475 Greifswald, Germany

**Keywords:** Roe deer, Microbiome, AMR, Shotgun metagenomics, Microbiome, Antimicrobial resistance

## Abstract

As an integral part of the global wellbeing, the health of wild animals should be regarded just as important as that of humans and livestock. The investigation of wildlife health, however, is limited by the availability of samples. In an attempt to implement a method with little invasiveness and broad areas of application, shotgun metagenomics were utilised to investigate the faecal microbiome and its antimicrobial resistance genes (AMRG) in roe deer. These genes can facilitate antimicrobial resistances (AMR) in bacteria and are therefore of increasing importance in global health. Accordingly, the abundance in potential vectors like wildlife needs to be assessed. The samples were additionally investigated for ESBL-*E. coli*, an antibiotic resistant pathogen of global concern, via cultivation. Twenty-seven hunt-harvested animals in Western Pomerania were sampled. This study is the first to our knowledge to describe the faecal microbiome of the European roe deer (*Capreolus capreolus*), providing insights into the bacterial and archaeal composition. Among the animals, the microbiome was mostly similar and showed a comparable composition to what has been reported in related species, with a ratio of 1.76 between *Bacillota* and *Bacteroidota*. The normalised abundance of AMR genes was found to be 0.035 on average, which is similar to other investigations on wild ruminants. Selective cultivation found no ESBL-*E. coli* in the animals. The prevalence of AMRG in roe deer of Western Pomerania was found to be in line with previous results. The use of shotgun metagenomics allowed for the simultaneous investigation of composition and AMR genes in the faecal microbiome of roe deer, which suggests it as a promising method for the health monitoring of wildlife. This study is the first to describe the prokaryotic assemblage in the faeces of roe deer and its differences to the microbiomes published on other cervids were discussed.

**Supplementary Information:**

The online version contains supplementary material available at 10.1038/s41598-025-93602-4.

## Introduction

While the health of humans and domestic animals tends to be under close surveillance, that of wildlife often goes undetected^1^. This is partially due to the limited recognition of its importance in earlier years, but also strongly caused by the poor accessibility to the desired object of study. The health of wild animals should be regarded as a matter of concern since it is not only closely knit to our own but also to that of the ecosystems surrounding us. Many of our domesticated animals are close ancestors to local wildlife and can therefore carry identical infectious agents^2^. These can often be transmitted in both directions, meaning that wild animals can be infected by farm animals or act as vectors for livestock^3^. In addition, zoonoses, linking the health of humans and animals, can follow the same paths and endanger both^3,4^.

As roe deer are the most abundant ungulates in middle Europe^5^, the species health is tightly knit with matters of nature conservation, economy and human as well as animal health. The high abundance of roe deer and its importance as game species facilitates interactions with humans and livestock through direct and indirect contact. Consequently, investigations of its health, especially in anthropogenic habitats, are of importance for understanding system dynamics and enabling informed decisions regarding management and control.

A striking example of the anthropogenic influence on our environment is the increasing detection of antimicrobial resistance genes (AMRG) in the microbial community inhabiting wild animals and natural habitats^6^. AMRG-carrying bacteria tend to increase relative to the anthropogenic input in the environment^7^, and it is suggested that wild animals facilitate the spread of AMRG-harbouring microorganisms^6^. Viewed as a serious threat to the shared health of humans, animals and the environment, further insight into the abundance and dispersal of AMR in the environment is imperative. The One Health approach was formed based around this understanding. Its goal is to unite researchers of different disciplines and establish a base of knowledge about these connections and mechanisms, as well as to keep the “shared health” intact, respectively replenish it^8^.

A 2023 review on the microbiota of Cervidae mentioned no study on the European roe deer^9^. This emphasizes the lack of insight into the microbiome of this species, which, due to its highly selective feeding style^10^, stands out from the Cervidae of Middle Europe. In the context of this study, gut microbiota are of additional interest, as they are the carriers of AMRG and their composition should consequently influence the class and abundance of resistances.

With the incentive of reducing the sampling and preparation effort needed to investigate the health of wild animals, we chose to simultaneously examine the microbiome and AMR genes of the European roe deer (*Capreolus capreolus*) through metagenomic shotgun sequencing. Samples were taken from the colon of hunt-harvested roe deer and additionally used to cultivate the intestinal microbiota on selective agar, searching for strains of extended-spectrum β-lactamase (ESBL)-producing *Escherichia (E.) coli*. These specific bacteria are receiving special attention because they are considered to be one of the major threats in the group of antibiotic-resistant bacteria^11–13^. The abundance of ESBL-*E. coli* and AMR genes, as well as the prokaryotic microbiome of 27 roe deer from Western Pomerania was investigated. To our knowledge, this is the first investigation on the faecal microbiome of *Capreolus capreolus*. We hypothetise a similar microbial composition to other wild cervids with some differences caused by the species’ selective feeding style. AMR abundance is expected to be low, as has been observed in wildlife in Germany^11^.

## Materials and methods

### Sampling

Sampling regions were located in the south of the city of Greifswald, and two hunting districts aided the sampling: Hegering Greifswald-Süd, dominated by fields and smaller wooded areas and the University Forest from the University of Greifswald, a partially disconnected forest area, which is used for forestry in parts and protected from economic use in others.

Animals were hunted within the legal hunting season by recreational hunters, which hold hunting licenses issued by the state. As of German law, hunted game is in the property of the land owner or tenant, who, in both regions, agreed to the sampling. For each of the two regions, hunters were instructed to reach out in case they hunted a roe deer and keep the intestines intact. Faecal matter from the rectum was then sampled within a maximum of 12 h. The samples were collected into 50 ml falcon tubes and swabbed with a Σ-Transwab^®^ (Medical Wire & Equipment Co Ltd, Corsham, UK) as soon as practicable to preserve bacteria in liquid amies transport media for cultivation. Swab samples were kept at temperatures under 10 °C in a fridge until further processing, while fecal samples were frozen at -20 °C within an hour of sampling.

With the pre-labelled tubes and sampling sheet, a form for additional information was provided. The hunter’s name, the date, location of the hunt and age and sex of the animal were documented on the sheet. Samples were taken between the 16th of April 2023 and the 1st of June 2023 (App. 1).

### Plating for ESBL *E. coli*

Swabs were stored at under 10 °C until they were streaked within a month on the chromogenic medium CHROMagar TM Orientation (MAST Diagnostica, Oldesloe, Germany), supplemented with 2 µg/mL cefotaxime (Alfa Aesar by Thermo Fisher Scientific, Kandel, Germany), which were incubated at 37 °C overnight. Simultaneously, the swab was incubated in LB-media at 37 °C overnight, supplemented with 2 µg/mL cefotaxime (Alfa Aesar by Thermo Fisher Scientific, Kandel, Germany). Afterwards, 100 µl of media were pipetted on CHROMagar TM Orientation, supplemented with 2 µg/mL cefotaxime (Alfa Aesar by Thermo Fisher Scientific, Kandel, Germany) and streaked. After a night of incubation at 37 °C, the agar plates were checked for red-purple colonies, which would indicate antibiotic-resistant *E. coli*. If respective colonies were found, one colony was repeatedly subcultured on CHROMagar TM until a pure culture was established.

### Metagenomic sequencing and analysis

Storage of fecal samples was at -20 °C. A total of 0.4 g of fecal matter from each deer were used to extract DNA after a month of storage, using the QIAamp Fast DNA Stool Mini Kit (QIAGEN, Hilden, Germany). The provided protocol was modified after Knudsen et al.^14^. Mainly, an additional extraction step using a TissueLyser (QIAGEN, Hilden, Germany) was added, followed by a higher lysis temperature of 95 °C and an increased proportion of proteinase K. Following the extraction, the DNA concentration was measured via the QuBit Fluorometer (Thermofisher Scientific, Waltham, MA, USA) with 1 µl of the extract. Illumina sequencing and shotgun metagenomic analysis were performed by SeqCenter (SeqCenter, Pittsburgh, USA). According to the company, sequencing was performed on an Illumina NovaSeq 6000 sequencer (Illumina Inc., San Diego, USA) after libraries were prepared using the Illumina DNA Prep kit and unique dual indices. Sequencing produced 2 × 151 bp paired-end reads.

Sequences were analysed for resistances through the default AMR + + pipeline and the MEGARes v3.0 database^15^. Within the AMR + + pipeline, low-quality bases and sequences were removed as well as host DNA. As output, counts of the AMR sequence hits on antibiotic class level were supplied as well as the corresponding genes and mechanisms. The raw data was further analysed through the METAXA2 pipeline^16^, to extract bacterial 16SrRNA sequences and return the corresponding number of reads in the dataset. Using the results of this pipeline, the normalized abundance of ARGs was calculated, following^17^. This process was done as described in^11^.

To assess the microbiome composition, the metagenomic analysis pipeline Kraken 2 was used with standard parameters (version 2.1.2) and the NCBI RefSeq database^18^.

### Data evaluation

All statistical analyses were performed in R^19^, using R Studio^20,21^. The α-diversity was classified by calculating the Shannon-Index^22^, Richness and Pielou’s Evenness^23^. No samples were excluded as outliers. Tests for significant differences were performed through a one-way analysis of variance by using the “aov” command. As β-diversity index, the Bray-Curtis dissimilarity was calculated between the samples and tested for significant differences though a PERMANOVA with 1000 permutations. The diversity measures and tests were calculated using the functions implemented in the package vegan^24^. Tests were performed on the microbial read data and the resistance gene abundance dataset. For testing, the samples were always grouped by location, age or sex. Dominance of a group was determined by highest abundance of reads or ARGs in a sample. To test for correlations between microbial read and resistance gene abundance in the samples, the Spearman rank correlation was calculated and tested for significance with the default Holm correction, using the psych package^25^. The analysis was performed on family level to keep most of the depth while producing a manageable output. With the same goal, families with less than 100,000 reads per sample were grouped in a subgroup of “others”. Visualization of the results was realised using ggplot2^26^. As all samples were sequenced to the same depth with a similar outcome in read numbers, no rarefaction normalization was performed.

## Results

### Sampling outcome

A total of 27 roe deer was sampled for this study. In an attempt to distinguish individuals with open from those with more forested habitats, samples were assigned to the dominant landscape type where the deer was hunted, as opposed to just differentiating by sampling region. App. 1 shows the sampling results including age and sex of the animal.

### Cultivation

No ESBL-producing *E. coli* were found in the 27 investigated samples, neither in the direct plating nor in the enrichment.

**Fig. 1:**
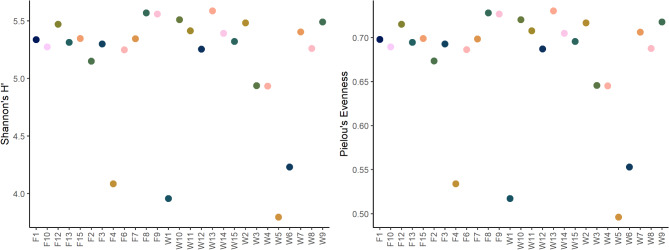
Shannon diversity and and Pielou’s evenness of bacteria within the investigated samples. Calculations were performed on genus level.

### Metagenomics

DNA extraction was performed on 27 samples. The average number of Illumina read pairs was 75,328,641.6 (± 7,852,905.3) with 22,357,186,368 (± 2,258,992,107) bases. Within the reads, identified as prokaryotic, some could not be assigned further than to domain level. In case of Archaea, 0.13% (0.13 ± 0.04%) of the reads (55,153 ± 40,704) could only be assigned to domain level. As for bacteria, 6.05% (6.05 ± 1.0%) reads (13,605,108 ± 2,820,730) could not be assigned further. In total, we identified 56 phyla, 563 families and 2,117 genera.

Alpha diversity was investigated on family and genus level, to be able to display possible differences between the levels. Shannon diversity and Pielou’s Evenness were noticeably lower in samples W1, W5, W6 and F4 on both, family and genus level. Apart from those four, the samples were homogenous in their alpha-diversity and evenness on both levels of analysis (Fig. 1). No significant differences in Shannon-diversity or evenness were found.

**Fig. 2:**
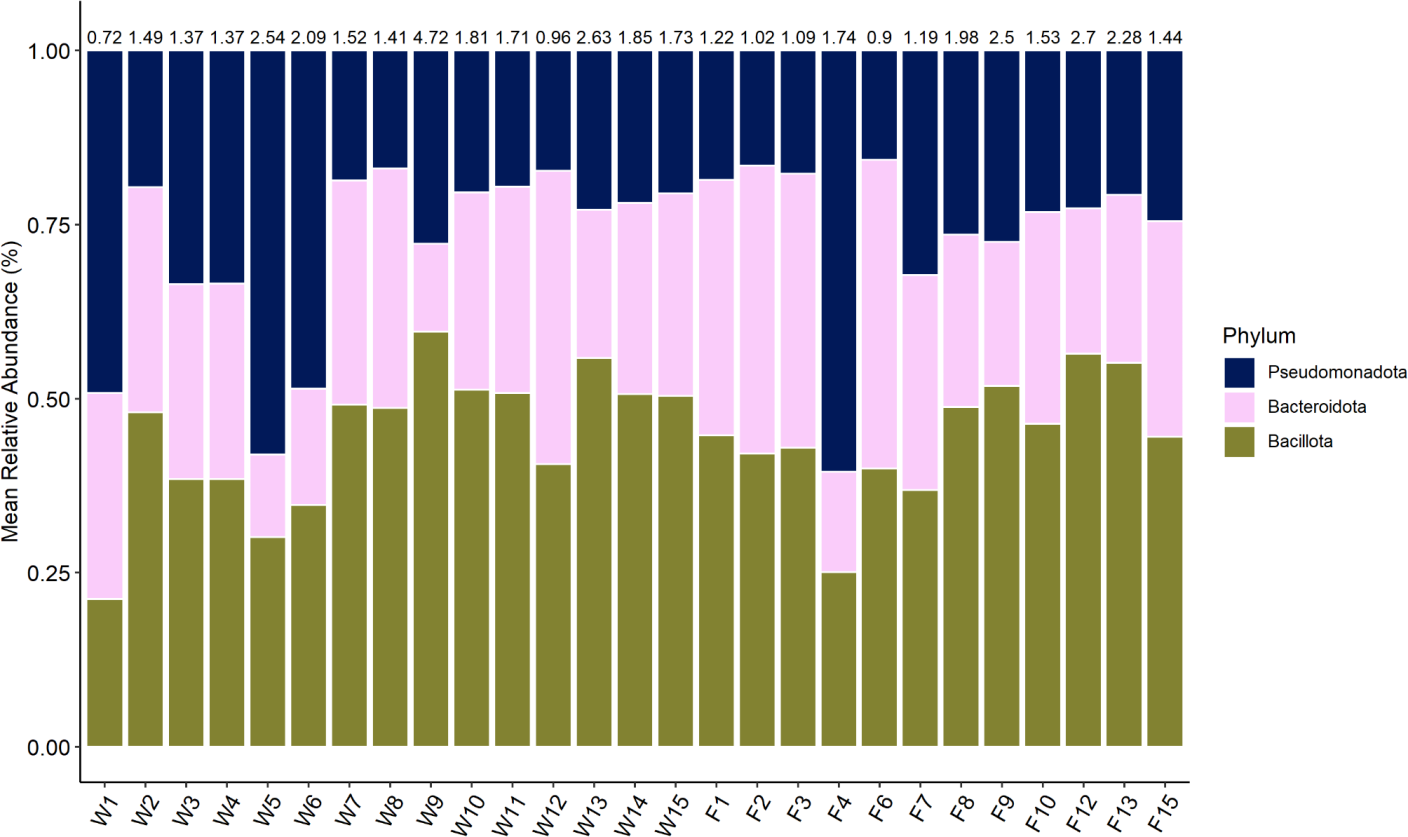
Mean relative abundance of the three most dominant phyla in each sample. Normalisation is based on only the abundances of those three phyla. Numbers above the bars show the ratio of *Bacillota* to *Bacteroidota*.

The overall dominant phyla were *Bacillota* (/*Firmicutes*) (37.5 ± 6.9%), followed by *Bacteroidota*(/*Bacteroidetes*) (24 ± 7.7%) and *Pseudomonadota*(/*Proteobacteria*) (23.2 ± 11.6%). These three most abundant phyla showed varying dominance in the individual samples. The ratio of *Bacillota* to *Bacteroidota* is 1.76 ± 0.8. W1, W5, W6 and F4, the samples with the lowest alpha-diversity all were dominated by 42.8–54.9% *Pseudomonadota*. Figure 2 shows the three most abundant phyla in relation and displays the ratio of *Bacillota* to *Bacteroidota* in each sampled animal.

**Fig. 3:**
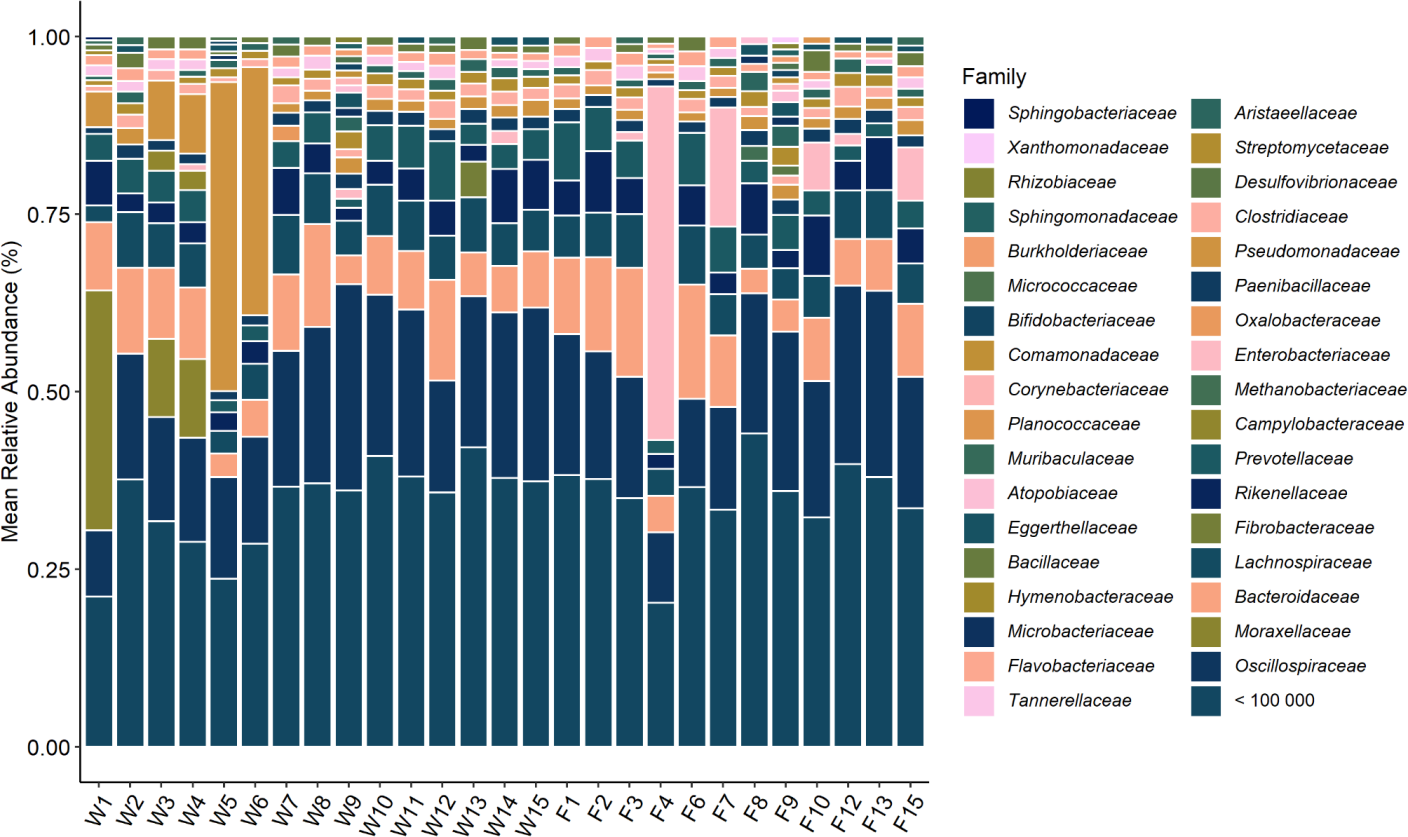
Mean relative abundance of the families occurring in each sample with a number of > 100,000 reads.

Analysis of the family level was performed on a dataset with all occurrences of less than 100,000 reads pooled into a group of “others”, to remove families of low abundance (Fig. 3). The full dataset can be accessed at the European Nucleotide Archive (Project No. PRJEB81356). On family level, *Oscillospiraceae* (18.9 ± 4.9%) showed the overall highest relative abundance, followed by the *Bacteroidaceae* (9.0 ± 3.7%), which showed a stable relative abundance in all samples. *Lachnospiraceae* (6.1 ± 1.5%) and *Pseudomonadaceae* (5.1 ± 10.1%) both were present in relative abundances larger than 5%. The dominant group of the individual samples varied. *Oscillospiraceae* were the dominant family in most samples but especially the four samples with low alpha-diversity varied. W1 was dominated by *Moraxellaceae* (33.8%), W5 and W6 by *Pseudomonadaceae* (43.5 and 34.5%, respectively) and F4 had a dominance of *Enterobacteriaceae* (49.9%) which was only the case in one other sample (F7, 16.8%). F6 was the only sample, where *Bacteroidaceae* showed the overall highest relative abundance (16.1%). The isolated high abundance of *Moraxellaceae* in W1 is mirrored on Genus level, where *Psychrobacter* (33.1%) dominate. This genus is only found in this sample. W5 and W6, both, show high abundances of *Pseudomonas* (43.4% and 35.0%, respectively). F4 is dominated by *Escherichia* (38.2%), which is present in every sample but at low percentages (0.001–4.9%).

**Fig. 4:**
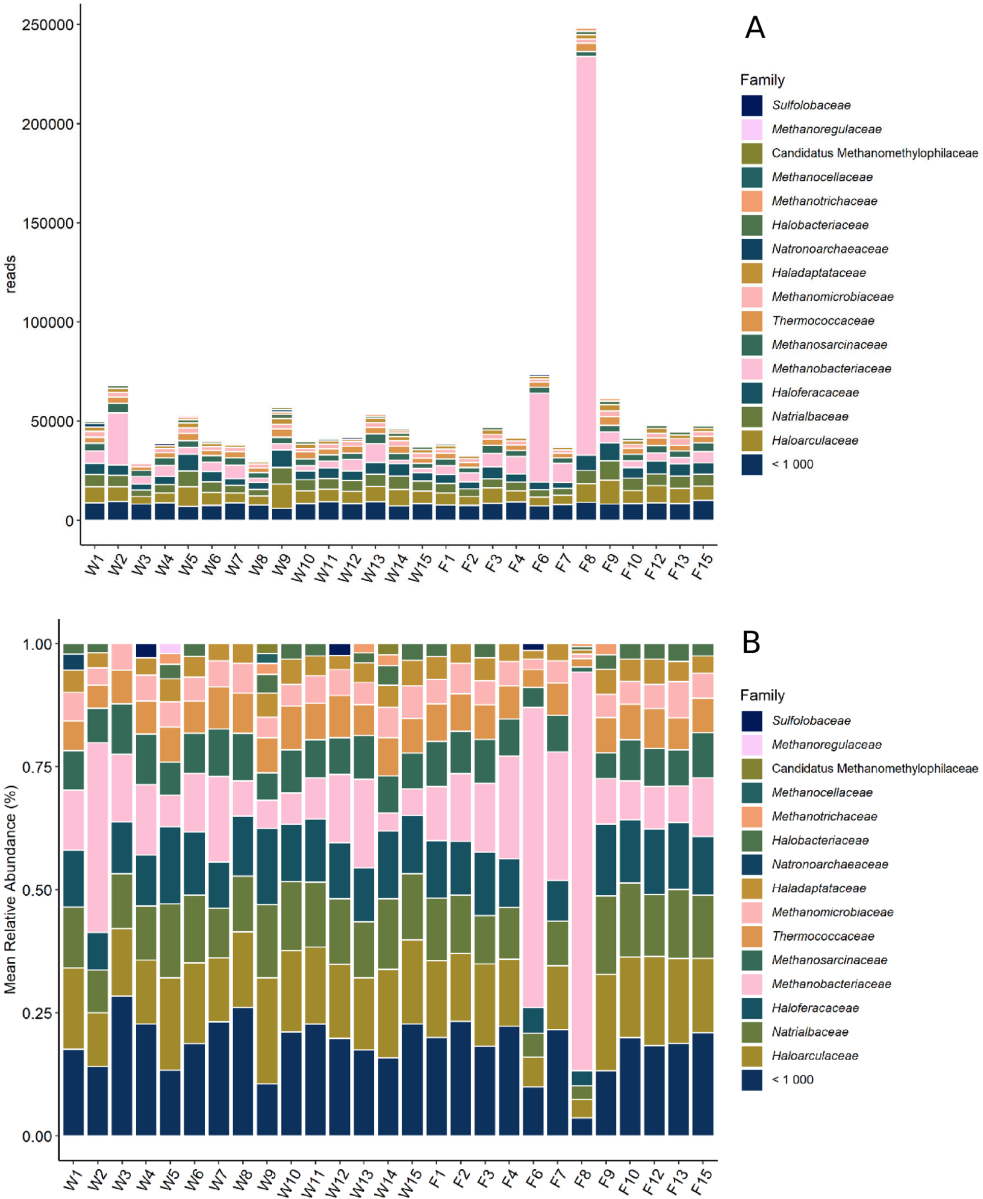
(**A**) Total abundance of archaeal family reads in each sample. (**B**) Mean relative abundance of archaeal family reads in each sample.

**Fig. 5:**
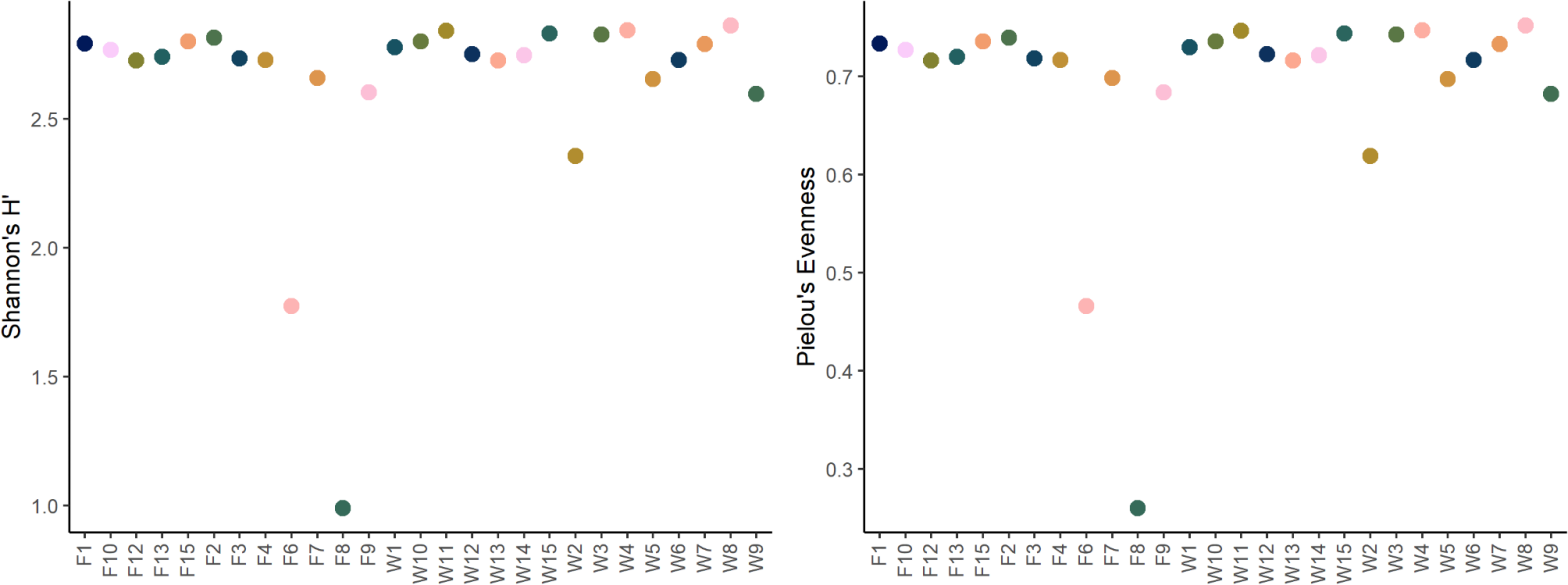
Shannon diversity and Pielou’s evenness of archaea within the investigated samples. Calculations were performed on family level.

Archaea were investigated on family and genus level. The number of archaeal reads in each sample was 45,123.4 (± 10,886.3) on average (Fig. 4A). The exception is sample F8, where 247,989 reads were detected. *Methanobacteriaceae* and *Haloarculaceae* showed the highest relative abundances (16.9 ± 17.4% and 15.0 ± 3.7%, respectively), they were present in all samples. *Natrialbaceae* (11.8 ± 3.0%) and *Haloferaceae* (11.3 ± 2.8%) also showed continuously high abundances in all samples. *Methanobrevicater* (14.3 ± 17.7%) was the most abundant genus in 17 samples, followed by *Thermococcus* (6.3 ± 1.4%) in the 10 other samples (Fig. 4B). On genus level it becomes clear, that most families are represented by multiple genera of small abundance. The ten most abundant genera only sum up to 42.4% of the reads, while on family level the ten most abundant families make up 82.8%. No significant differences in diversity (Fig. 5 (alpha diversity)) were found in relation to the investigated factors. The Shannon Diversity and Pielou’s evenness between the samples were found to be relatively homogenous, apart from samples W2, F6 and F8. All three of these samples show an increased number of reads, caused by the high abundance of *Methanobacteriaceae*, which appear as *Methanobrevibacter* on genus level.

**Fig. 6:**
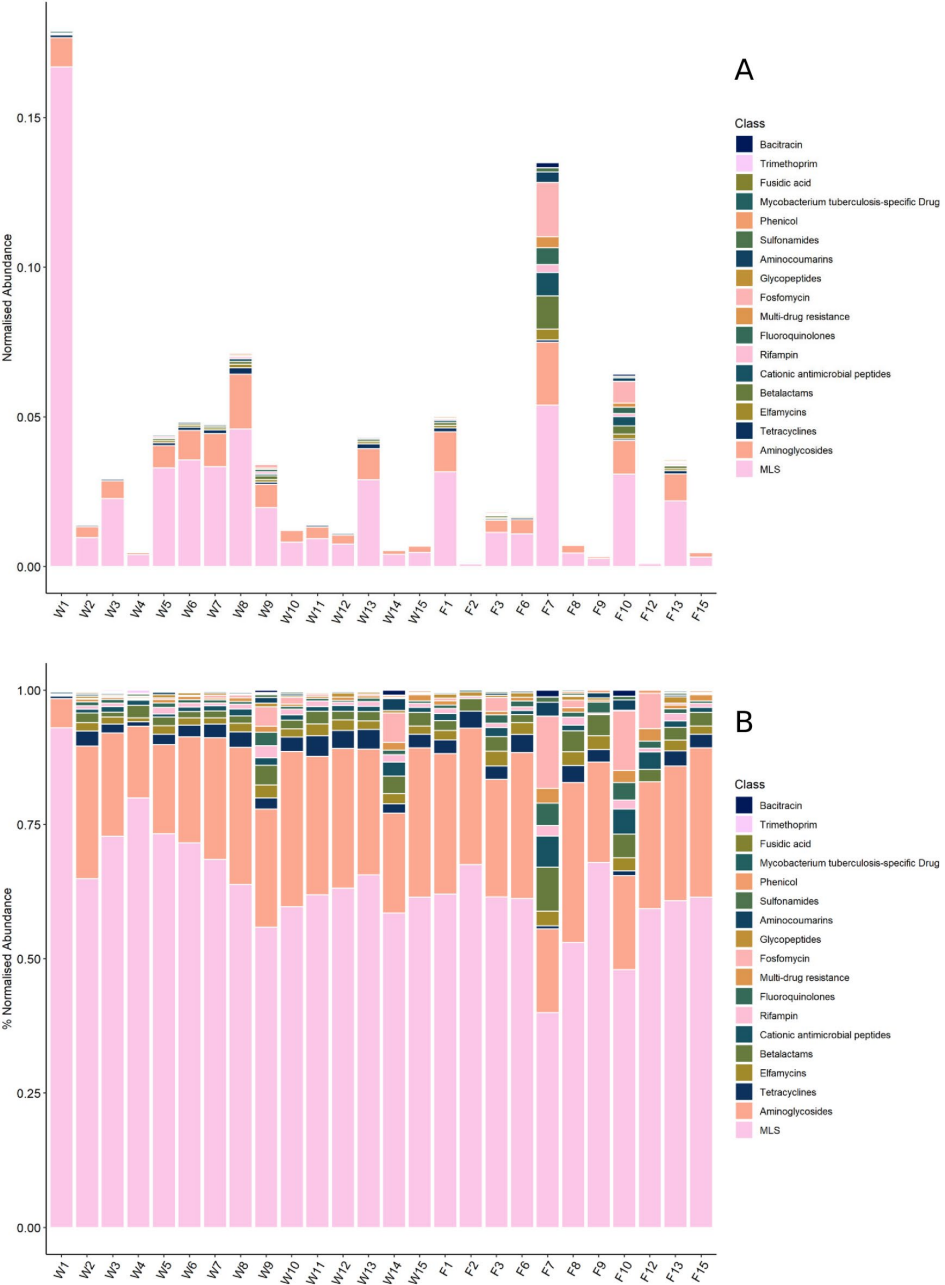
(A) Total normalized abundance of resistance gene reads in each sample. (B) Mean relative normalized abundance of resistance gene reads in each sample.

The normalized total AMR abundance (App. 2) was on average 0.035. The total normalised abundance of resistances varied greatly between the samples (Fig. 6A). Most abundant were resistances against the class of macrolides, lincosamides and streptogramines (63.7 ± 10%), represented mostly by the gene MLS23S (61.7 ± 11.8%) (App. 3). This was the most abundant resistance in all samples, followed by aminoglycosides (22.2 ± 5.6%). The associated genes of noticeable abundance were A16S (13.9 ± 5.4%), rrsC (2.3 ± 1.4%), rrsH (2.1 ± 1.6%) and rrsA (1.2 ± 1.1%). Fosfomycin resistances (1.9 ± 3.5%) were only detected in 19 of the samples but showed up in relatively high abundances of up to 13.4% in some (F7). Tetracylines were present in a relative abundance of 2.3 ± 5.3% (Fig. 6B). The abundance of all resistance genes found is shown in App. 4. No significant difference between the samples in relation to the investigated factors of age, sex or hunting location could be identified.

According to the investigated factors, the β-diversity revealed no significant differences between the groups. (App. 5)

**Fig. 7:**
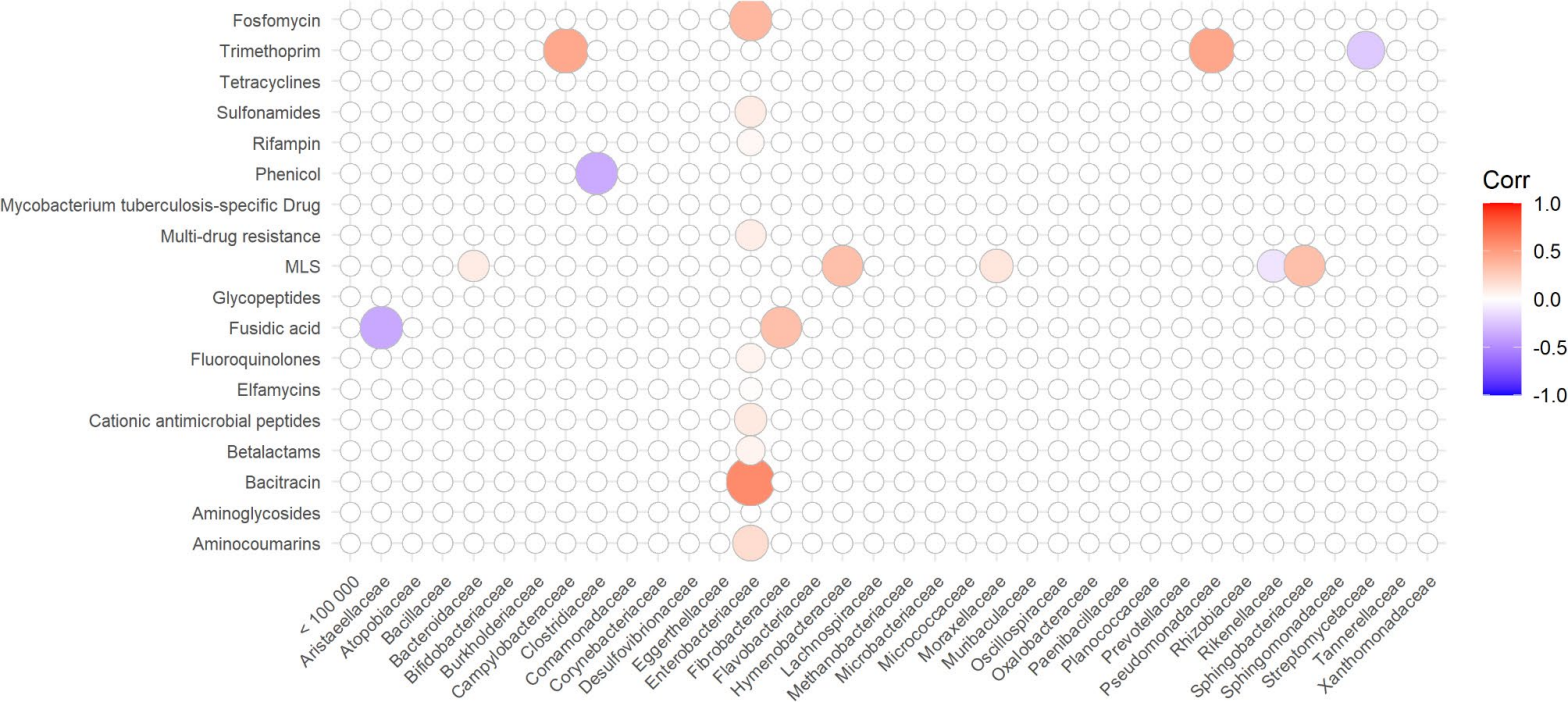
Spearman-rank-correlation of the abundance of bacterial families and resistance gene reads. Only correlations with a significance of > 0.05 are displayed.

A spearman-rank correlation showed several significant moderate co-occurrences of bacterial families and antimicrobial resistance reads (Fig. 7). The most correlations were found between the family of *Enterobacteriaceae* and several antimicrobial resistances, more precisely bacitracin (ρ = 0.59, *p* < 0.001). *Pseudomonadaceae* and *Campylobacteraceae* had a correlation to trimethoprim (ρ = 0.46, *p* = 0.002 and ρ = 0.45, *p* = 0.01, respectiveley).

## Discussion

### Microbiome - Bacteria

The low variability of alpha diversity and evenness points to a stable microbiome among the different animals tested, which seems not to be influenced by sex or age of the host animal. Similarly, no significant differences in β-diversity could be observed between the microbiomes of the animals, sampled in the two different hunting regions. This is an indicator that the environmental characteristics of the two regions are similar, since other studies found differences in the faecal microbiota of mule deer to be influenced by geography^27^. However, some samples showed diverging compositions, these datasets will be discussed later.

Our finding of that *Bacillota* (/*Firmicutes*), *Bacteroidota* (/*Bacteroidetes*) and *Pseudomonadota* represent the most abundant phyla is in accordance with literature on other cervids^9,28–30^. In studies of the gastrointestinal microbiome, the relation of *Bacillota* to *Bacteroidota* has been used as an indicator for the metabolic efficiency of the microbiome. A higher ratio in cervids implies a maximised energy extraction from the cellulose-rich feed^31^, since *Bacillota* have the ability to decompose cellulose to volatile fatty acids, while *Bacteroidota* tend to have a regulatory effect on the carbohydrate metabolism^32^. The average ratio of 1.76 ± 0.8 indicates the possibility of increased energy harvest from cellulose in the sampled animals. Samples, where the ratio was smaller than one could originate in animals which foraged on carbohydrate-rich foods like crops. Yet, not all species within a phylum will perform the same metabolic functions and deviations from the average could also result from factors like the general health of the animal or gastrointestinal imbalances.

The variation on phylum level has been reported to be low in the microbiome of mule deer^27^, which is supported by the findings of this study. While most authors report a dominance of *Bacillota* and *Bacteroidota*^9,28–30^ in the faecal microbiome of the investigated cervid species, greater differences appear if the microbiota are compared on lower phylogenetic levels. *Oscillospiraceae* (/*Ruminococcaceae*) are reported in most cervids^9,28–30^ and make up the most abundant family of this investigation. They are associated with digestion of fibre, specifically cellulose^28,30^, which is in line with the dietary habits of cervids. *Bacteroidaceae* or related groups have also been found in mule deer (*Bacteroidales*^27^), , Siberian roe deer (*Bacteroides*^30^), and white lipped deer^9^. They are associated with monosaccharide utilisation and plant polysaccharide decomposition^30^, so their abundance probably stems from their functional role in the gastrointestinal system. *Lachnospiraceae*, again, can ferment cellulose and fibres^30^ and have also been found in several other cervids^9,29,30^ apart from the roe deer, investigated in this study. *Lachnospiraceae* as well as *Oscillospiraceae* belong in the order of *Eubacteriales*, which is often mentioned as highly abundant order in the literature^27,29,30^. Thereby, the abundance of these families in the samples of this study is supported by other investigations. Another family which appeared regularly in the microbiomes of the roe deer were the *Pseudomonadaceae*. This family was especially abundant in the samples which showed deviations in the diversity parameters but also showed up in percentages higher than 5% in samples W3 and W4. This is an unusual finding, since *Pseudomonadaceae* are generally not mentioned as typical inhabitants of the faecal microbiome. The presence of this family could indicate dysbiosis, which may have been the result of a gastrointestinal infection or another kind of disruptive event. Since no clinical investigation was carried out, this remains open. Pacheco-Torres et al.^9^ conclude the persistence of a core bacterial community in the individuals of one species, which is in line with the findings of this study.

An exception are the four samples which show deviating characteristics in terms of diversity as well as composition. The dominant family of W1 are *Moraxellaceae* which are mostly represented by *Psychrobacter*, a genus made up of environmental psychrophiles as well as inhabitants of the animal microbiome^33^. In another study, *Moraxellaceae* were found in white tailed deer^29^, represented by the genus of *Acinetobacter*, which is also the case for most of the other *Moraxellaceae* in this investigation. Some species of *Psychrobacter* are found in mammalian guts and a part of them can cause disease^33^. The dominance of *Psychrobacter* in sample W1 could point to a dysbiosis in the gut microbiome, caused by pathobiontic *Psychrobacter*. Further insight into the specific strain would be intriguing, since the genus was, so far, mostly detected in guts of humans and marine mammals^33,34^. Samples W5 and W6 were dominated by *Enterobacteriaceae*, which have also been found to be common in white tailed deer^29^. In the case of this study, the corresponding genus is *Pseudomonas*. This genus is not typically reported as relevant in the microbiomes of cervids. Zhao et al.^35^ mentioned it in Chinese forest musk deer and they have been reported in the rumen of healthy sheep^36^. In the giant panda, *Pseudomonas* is the dominant strain of the gut microbiome and is involved in lignin-degradation^37^, which would be of advantageous for deer, too. Another possible explanation is the infection of the animals with pathogenic strains, which are clinically relevant and can carry an array of AMR^36^. The dominance of *Enterobacteriaceae* in sample F4 is reflected at genus level, in the form of *Escherichia*. High abundances of *Enterobacteriaceae* have also been found in white tailed deer^29^. The genus *Escherichia* is common in the gut of mammals and can sometimes be pathogenic^9^ and often carries AMR of high clinical importance^11^. Its increased abundance in this sample could point to a dysbiosis. All four of these deviating samples are caused by bacteria within the family of *Pseudomonadota* and show an increased number of reads in relation to the other samples. This could point to a competitive advantage of these bacteria, which led to increased growth and thereby abundance. Deviations like these could also stem from changes within the samples, that occurred during the storage after sampling. Carroll et al.^38^, investigated faecal samples during a storage time of 24 h at room temperature to gain insight into possible community shifts and found no significant differences in composition and diversity to samples which were frozen immediately.

Generally, the results of this study compare well to those on other cervids and open the door to further investigations on individuals from varying regions and conditions.

### Microbiome - Archaea

As observed with the bacteria, archaea did not vary significantly between the roe deer and seem to build a rather homogenous microbiome, again with some deviating samples. Comparisons to other literature is insofar complicated as little publications on the full archaeal gut microbiome of cervids exists, most studies focus on the (rumen) methanogen community^39,40^ or the human archaeome^41^. It is striking, that *Haloarchaea* like *Haloarculaceae*, *Natrialbaceae* and *Haloferaceae* made up some of the most abundant families, since this group contains mainly halophiles, which would not be expected in the microbiome of a terrestrial mammal. Nevertheless, the detection of halophilic archaea in animals is common, although the reasons are not yet understood^40^. In humans, the occurrence of halophiles was hypothesized to be connected to the consumption of salty foods^40^. Accordingly, these organisms could be the result of the use of mineral licks, which are supplied as part of management practices to wildlife in Germany^42^. This could be an interesting line of investigation for further metagenomic analyses of the microbiome of wild animals. Similar to this study, Han et al.^28^ also found a high abundance of *Methanobrevibacter*, when investigating Methanogens in Siberian roe deer. The genus is also prevalent in the gut microbiome of humans, where it is correlated with the ingestion of carbohydrates^41^, cattle and sheep^39^. Apart from this genus, further methanogens, like *Methanosarcina*, were found in lower abundances in their samples, which is in congruence with findings in several species of mammals^40^. It is still unclear, if methanogens are harmful to their host because of the methane production or if they might even increase feed utilisation through mechanisms like hydrogen reduction, which benefits bacterial polysaccharide fermentation^39,43^. Samples W2, F6 and F8 showed an increased abundance of *Methanobacteriaceae*, which also presents in the lowered diversity indices. Notably, these diverging samples have no overlap with the ones standing out in regards to bacterial diversity. This points towards a biological reason, such as a different kind of feed, for the variation, rather than a methodological artefact. *Thermococcales*, the second most abundant order is known as anoxic, thermophilic inhabitant of hydrothermal habitats^44^ but has also been mentioned once as abundant in the microbiome of marmots^45^. The functional or ecological reason for its abundance in the guts of mammals, while being considered an environmental extremophile in pan-genomic studies could be of interest for further exploration of the order. Transitions from environmental lifestyles into the gut of animals has been proposed for multiple archaeal lineages and could be applicable to this group^46^. The low variation found between the archaeal assemblages of the animals is in line with the results of Youngblut et al.^47^, who found, that host phylogeny had a higher influence on the composition of gut archaea, than diet did. Looking at the samples with deviating characteristics, *Methanobrevibacter* makes up a large part of the reads, which is plausible in the context of other investigations^43^, but the reason behind the differing composition remains unclear. Since the archaeome of the European roe deer has not been investigated before, direct comparisons cannot be drawn. Within the context of data on other cervids, the results of this study are plausible and show some intriguing details, like the abundance of halophilic groups and the order *Thermococcales*, which inspire further research. This publication can hopefully become a point of comparison for future research of the matter.

### Resistome

The normalized AMR abundance was slightly higher than in another study on two roe deer in the same state, where an average of 0.0305 was found^11^. In dairy calves, an abundance of 0.77–5.14 normalized total antimicrobial resistance genes were found^48^, which supports the assumption, that wildlife carries a lower AMRG burden than livestock^49,50^.

Some prevalence of AMR genes can be expected within natural populations of bacteria^51^ and AMR *E. coli* have been found in wild deer without direct exposition to antimicrobials^52^. Even so, the abundance in wild animals increases with their connection to human activities^53,54^ and AMR-carrying bacteria seem to be brought into the environment through anthropogenic sources like contaminated water^53^ or manure^55^. Animals in close association to humans have a higher risk of being infected with AMR bacteria, as do omnivorous and carnivorous species – probably due to their rank in the food web^53^. But wild deer too, especially when in contact with feeding stations^55^ or living in a herd lifestyle^54^, are part of the wildlife reservoir and AMR-carrying bacteria have been found in roe and red deer in Scotland^52^, as well as in red deer in Portugal^20^ and white-tailed deer in the US^55^.

The heavy variation in normalized resistance abundance could be rooted in various factors. On biological levels, influences like the exposure to antimicrobials or AMRG contaminated environments can be a reason, although no significant effect of the sampling location could be found. The intestinal microbiome is impacted by an animal’s habitat and so is the connected resistome^6^. Different microbiome compositions could harbour varying prevalences and classes of AMRGs. This will be further explored later, by correlating the microbial composition and AMR classes. Lastly, biases can emerge during extraction and sequencing of the DNA. Since all samples underwent the same procedures though, at least the DNA of microorganisms of the same type should appear in comparable abundances in the dataset.

Macrolide, lincosamide and streptogramin (MLS) resistances dominated throughout the dataset, which is reflected in a similar study on two roe deer by Homeier-Bachmann et al.^11^, where the two investigated roe deer (Western Pomerania, Germany) showed a normalized abundance of 28.2% MLS resistance genes. Dias et al.^20^ found macrolide, lincosamide and streptogramin B (MLSB) resistances to be especially frequent in red deer (Portugal) adjacent to livestock farming. Their investigation was based on a qPCR assay though, which limits the comparability. In relation to these studies, the MLS resistance gene abundance of over 50% in the animals within this study stand out. Regarding Aminoglycoside resistances, an abundance of 7.55% was found by Homeier-Bachmann et al.^11^ and Osińska et al.^54^ were able to detect streptomycin resistance genes in seven out of eight *E. coli* isolates from roe deer (Poland). Fosfomycin was found at an abundance of 1.2% by Homeier-Bachmann et al.^11^, which is similar to the results of this study. While this study found only low abundances of β-lactam resistance genes, several other investigations mentioned resistances against this class or corresponding antibiotics, which is probably due to the high clinical importance of β-lactam resistances^56^. Osińska et al.^54^ found ampicillin resistance in all investigated *E. coli* isolates from roe deer, Elsby et al.^52^ reported phenotypical resistance against cefpodoxime and ciprofloxacin in *E. coli* isolates from the faeces of Scottish wild deer (6.5% and 0.3%, respectively) and Dias et al.^20^ detected ampicillin resistance in 12% of *E. coli* isolates from red deer (Portugal) and an abundance of 10^− 5^ β-lactam resistance genes, normalized against the abundance of 16SrRNA genes. The lack of standardisation when investigating AMR results in a lack of comparability between studies. Especially the normalization of reads varies between studies. The method used within this study was the same as in Homeier-Bachmann et al.^11^, which allows a direct comparison of abundances in contrast to those reported by Dias et al.^20^. Homeier-Bachmann et al.^11^ found a normalized abundance of 7,55% β-lactam resistance gene reads, which is considerably higher than the 2.4 ± 2.1% in this study. Several studies mentioned tetracycline resistance as highly abundant in the investigated deer^11,20,52,54^. This observation could not be confirmed by this study, where normalized resistance gene reads of tetracycline made up only 2.3%. No ESBL-*E. coli* could be isolated from any of the samples, which is in accordance with similar investigations in Germany. Plaza-Rodriguez et al.^49^ found a prevalence of 2.3% in roe deer and Homeier-Bachmann et al.^11^ 1.1% in wild ruminants.

There is no recognizable pattern in the comparison between the results of Smoglica et al.^6^ with the ones of this study. The correlations between resistance classes and bacterial groups did not yield similar results. Most correlations found by Smoglica et al.^6^ related to tetracycline resistance genes, which were rare in this study. The comparability between the studies is limited by the differing methodology. Smoglica et al.^6^ used 16 S rRNA gene sequencing to characterize the microbial community and a qPCR approach to find a panel of six specific AMRGs. This study found a co-occurrence between the family of *Enterobacteriaceae* and bacitracin resistance. As bacitracin is effective mostly against gram-positive bacteria^57^, *Enterobacteriaceae* are not affected by the compound. The correlation might be a statistical artefact, caused by the increased abundance of *Enterobacteriaceae* in certain samples. Another possible explanation would be a co-occurrence of certain bacterial groups, carrying bacitracin resistance genes, with the family of *Enterobacteriaceae*. *Campylobacter*, a genus within the *Campylobacteraceae*, was considered to be intrinsically resistant to thrimethoprim but has also been shown to acquire *dfr* genes, facilitating the resistance^58^. The gene has also been found in *Pseudomonas spp*. from raw milk, although it was only shown in one isolate^59^. Since these genes are part of the MEGARes database^15^, it is possible that the bacteria present in the microbiome carried those AMRGs.

### Limitations

6.05% of the bacterial and 0.13% of the archaeal reads remained unassigned. This can be the result of multiple technical and methodical causes. Part of the reads probably belong to the microbial dark matter (MDM), which cannot be assigned to any further group because the particular organism has not been described or its genome uploaded into the database yet^60^. Since the use of shotgun sequencing produces untargeted reads, the 16 S rRNA gene, the usual identification marker of prokaryotes, is not necessarily part of the sequencing result. Therefore, the taxonomic assignment by Kraken2 is based on the mapping of short Illumina reads to the full genomes within its database and to avoid overclassification, the lowest common ancestor is chosen, if reads map to several genomes^18^.

The sampling of hunt-harvested roe deer introduces a certain bias, since hunters in Germany are instructed to focus their activities on the weakest animal of a group Therefore, the body condition of the sampled animals might be lower than average for a population. At the time of year where sampling took place, the hunting target is male yearlings while the season for does is closed. This influences the sample distribution along the population. Consequently, the results of this investigation are mostly based on those male yearlings, which should be considered in comparing this data. A sample size of 27 individuals may be too small to accurately represent the species’ microbiome and abundance of AMR and may influence statistical power. More biases can emerge from the method of DNA extraction^61^ and sequencing. Sachez-Cid et al.^62^ found a greater influence of sequencing depth than of the DNA extraction on the bacterial richness. Additionally, the abundance of host-DNA can overshadow the true microbial diversity in microbiomes and lead to a decreased detection of species^12^. Since the samples in this study were sequenced with a depth of 75,328,641.6 (± 7,852,905.3) read pairs, the possibility to detect rare species remains^63^.

## Conclusions

The use of shotgun metagenomics allowed for the simultaneous investigation of microbial composition and AMR genes in the faecal microbiome of roe deer. This study is the first to describe the prokaryotic assemblage in the faeces of roe deer and some differences to the microbiomes published on other cervids were discussed. In relation to other comparable studies, the abundance of AMR genes was similar. No ESBL-*E. coli* could be detected, which is in line with the expected abundance in Western Pomerania. Despite the relatively low numbers of detected resistance genes, these consistent findings in wildlife should be reason for implementing further action against the emergence and spread of AMR.

In the future, this approach could be used for monitoring purposes in the health of less accessible species and to deepen our understanding of the microbiome composition of wild animals. Larger future studies could make use of this method for analysing variation in space and time, as well as the wildlife-livestock interface.

## Electronic Supplementary Material

Below is the link to the electronic supplementary material.

Supplementary Material 1

Supplementary Material 2

Supplementary Material 3

Supplementary Material 4

Supplementary Material 5

Supplementary Material 6

## Data Availability

The datasets generated and analysed within this study are available in the European Nucleotide Archive repository with the primary accession code PRJEB81356.
