# Supplementary figures and images for: Metagenomic analysis of the faecal microbiota and AMR in roe deer in Western Pomerania
Source: Sci Rep. 2025 Mar 18;15:9288. doi: 10.1038/s41598-025-93602-4 (PMC11920406; doi:10.1038/s41598-025-93602-4)

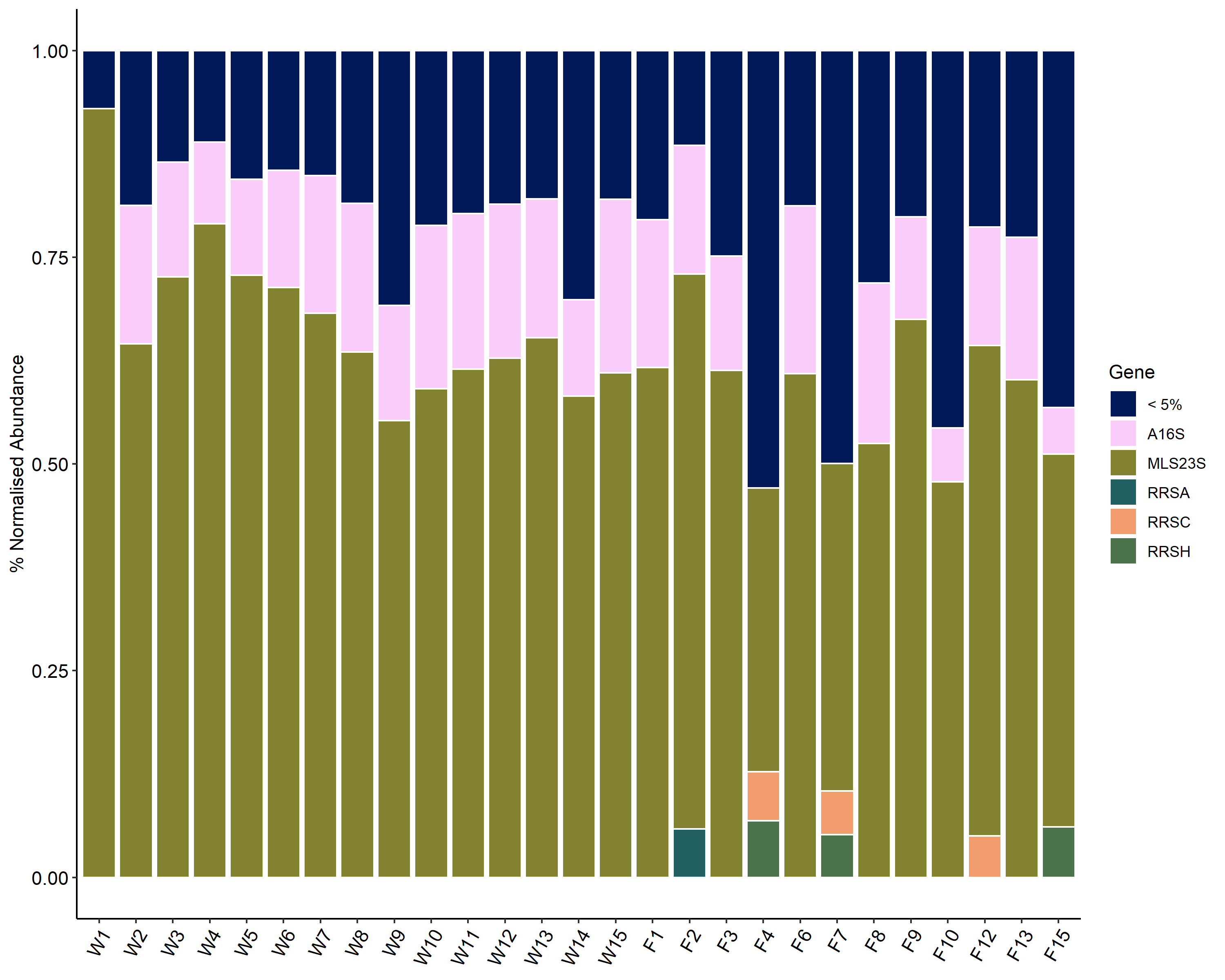

Supplement: Supplementary file 3 — Supplementary Material 3 [file 41598_2025_93602_MOESM3_ESM.tiff]
