# Supplementary material for: Metagenomic analysis of the faecal microbiota and AMR in roe deer in Western Pomerania
Source: Sci Rep. 2025 Mar 18;15:9288. doi: 10.1038/s41598-025-93602-4 (PMC11920406; doi:10.1038/s41598-025-93602-4)
Supplement: Supplementary file 6 — Supplementary Material 6 [file 41598_2025_93602_MOESM6_ESM.docx]

Appendix Information

App. 1: Sampling results

App. 2: Normalized AMR abundance

App. 3: Mean relative normalized abundance of resistance gene reads in each sample. Genes with a relative abundance of less then 5% are summarised under “< 5%”.

App. 4: Normalized and relative abundance of resistance gene reads

App. 5: Bray-Curtis distance of samples on family level
